# Achievements and Visibility of Scientific Publications of All Peruvian Medical Schools: A 5-Year Scientometric Analyses

**DOI:** 10.1155/2022/9097379

**Published:** 2022-04-25

**Authors:** Frank Mayta-Tovalino, Josmel Pacheco-Mendoza, Jesus Bardales-Garcia, Juan Alvitez, Abigail Temoche, Roman Mendoza, Arnaldo Munive-Degregori

**Affiliations:** ^1^Vicerrectorado de Investigación, Universidad San Ignacio de Loyola, Lima, Peru; ^2^Unidad de Investigación en Bibliometría, Universidad San Ignacio de Loyola, Lima, Peru; ^3^Posgraduate School, Master of Science with Mention in Public Health, Universidad Nacional de Cajamarca, Cajamarca, Peru; ^4^Academic Department, Faculty of Medicine, Universidad Nacional Federico Villarreal, Lima, Peru; ^5^Posgraduate Department, Faculty of Dentistry, Universidad Nacional Federico Villarreal, Lima, Peru; ^6^Postgraduate Department, Faculty of Letters and Human Sciences, Universidad Nacional Mayor de san Marcos, Lima, Peru

## Abstract

**Objective:**

To evaluate the bibliometric profile of the national academic production of public and private medical schools in Scopus after the publication of a new university law (2015–2020).

**Methods:**

A descriptive bibliometric study was conducted. The secondary data corresponding to the 42 Peruvian medical schools that had scientific publications in Scopus from 2015 to 2020 were evaluated, and the Affiliation ID of each Peruvian institution was considered. Data were analysed using SciVal.

**Results:**

The total production of the Peruvian medical schools was 5406 manuscripts (100%), of which 3018 (56%) were included in the ‘clinical medicine' category. The total production of publications in Peru from 2015 to 2020 was 21597 manuscripts, of which medical publications accounted for 7950, with only 5406 manuscripts belonging to the Faculties of Medicine of Peru. The *Peruvian Journal of Experimental Medicine and Public Health* had published the highest number of manuscripts (500 articles), with an approximate publication citation of two.

**Conclusion:**

There was a constant increase in the scientific production of Peruvian medical schools from 2015 to 2020. Additionally, it was found that medical publications contribute the most to the total scientific production of Peru.

## 1. Introduction

Scientometrics consists of the application of different statistical methods to analyse results of scientific research indexed in databases such as Embase, Scopus, Medline, and Web of Science. This area measures the scientific productivity of institutions, especially in the field of medicine [1].

Bibliometric studies generally analyse the quantitative characteristics of scientific publications to obtain findings in relation to scientific communication. This type of research enables drawing contrasts between countries, affiliations, and/or authors. Consequently, bibliometrics is the numerical analysis of scientific publications in a certain period, field, or institution, and the collaborations between them [2].

Scientometrics analyses study the impact of scientific production in an area of knowledge, such as collaborative networks that help identify dynamics between authors, countries, and institutions [3, 4]. In this sense, it is important to know the activities related to scientific publication in the faculty of human medicine and quantify their impact based on number of citations. Medical schools play an important role in the creation of knowledge based on scientific evidence, especially for optimal decision-making [5].

However, a lack of methodological uniformity can be observed in the evaluation of scientific production by medical schools, which may directly impact their reputation. For example, when evaluating the scientific production of medical schools in Europe, different bibliometric data have been used to track the amount of international collaboration, volume, and increases in research production, integrating different bibliometric techniques that could provide a more nuanced and accurate interpretation of the research output of medical schools [6].

Thus, this research was aimed at determining the scientometric profile of the national academic production of all Peruvian medical schools in Scopus.

## 2. Methods

### 2.1. Study Design

This scientometric study was of retrospective, descriptive, and observational design. All medical schools in Peru that had a Scopus ID were evaluated, which corresponded to 42 faculties. The scientific production of these faculties was evaluated between 2015 and 2020 (Table 1)

### 2.2. Search Strategy

The search strategy was developed based on the institutional profiles (Affiliation ID) of Peruvian universities that had a school of human medicine. Afterwards, the profiles found in the Scopus database were exported to a CSV file, which was then imported into the SciVal tool to proceed with the bibliometric analysis. This analysis included indicators such as scientific production by sub-subject area, total scientific production, type of collaboration, scientific production by university, and indexed journals with the highest publication of Peruvian medical manuscripts.

### 2.3. Medicine School Selection

All Peruvian colleges were evaluated according to the licensing pattern issued by the regulatory entity SUNEDU (National Superintendency of Higher Education). The following selection criteria were applied:

Inclusion criteria are as follows:

- public Peruvian medicine schools
- private Peruvian medicine schools
- public and private Peruvian medicine schools with institutional licensing by SUNEDU

Exclusion criteria are as follows:

- Peruvian medicine schools that do not have Scopus registration

### 2.4. Statistical Data Analysis

A descriptive statistic of the scientific production of all the evaluated medical schools was obtained and tabulated in Excel. Bibliometric analysis was performed using the SciVal tool from Scopus.

## 3. Results

### 3.1. Scholarly Output of Peruvian Medical Schools

The total production of the Peruvian medical schools was 5406 manuscripts (100%), of which 3018 (56%) were included in the “clinical medicine” category. In contrast, the “basic medicine” and “health sciences” categories were represented by 36% and 34%, respectively. Finally, only 1% of the investigations were in the “medical biotechnology” category (Table 2).

### 3.2. Scientific Production of All Medical Schools in Peru

Of the 42 Peruvian universities with a faculty of medicine, the leading institutions were Universidad Peruana Cayetano Heredia, Universidad Nacional Mayor de San Marcos, and Universidad Peruana de Ciencias Aplicadas, with 2172, 975, and 705 manuscripts, respectively. In relation to other institutions, a progressive increase was observed from 2015 to 2020 (Table 3).

### 3.3. Top 10 Scientific Journals which Publish Manuscripts from the Medical Schools of Peru

The Revista Peruana de Medicina Experimental y Salud Publica had the highest number of manuscripts at 500, with an approximate publication citation of two. *The American Journal of Tropical Medicine and Hygiene* was in the second place with a total of 130 published manuscripts and a higher average publication citation of 7.2. The *Revista de Gastroenterología del Peru* was in the third place with 115 published manuscripts, although its average publication citation (0.7) was one of the lowest (Table 4).

### 3.4. Top 10 Authors Who Published Manuscripts from the Medical Schools of Peru

The most productive authors that exceeded 100 publications in this category were Miranda, Juan Jaime; Bernabe-Ortiz, A; and Mejia, Christian R, from the Universidad Peruana Cayetano Heredia and Universidad Continental, with 213, 126, and 110 published manuscripts, respectively. The first two authors had an average of 44 and 56.8 citations per publication, while the third author had 2.6. Finally, the authors with the best *h*-index were Gilman, Ribert Hugh; Gotuzzo, Eduardo H.; García, Héctor Hugo; and Hernandez, Adrian V., with an *h*-index of 55, 49, and 48, respectively (Table 5).

## 4. Discussion

Scientometric analysis is a conglomeration of statistical methods used to quantify scientific production. Bibliometric mapping provides an excellent method for visualizing publication and citation information. This allows information to be represented in ways that make relationships objective and easy to understand [7].

Citation analysis of scientific articles is a method used to evaluate the quality of published scientific articles. The number of citations is generally correlated with the quality of the research. Citation analysis matches well with other indices of scientific recognition; therefore, it is frequently used to evaluate the academic production of a certain subject, author, or institution. Therefore, this bibliometric study can help researchers understand published scientific information and help doctors make decisions [8–12]. The present study used different bibliometric methods to measure factors such as scientific production, collaboration, and citations. However, it may include undue biases since it uses secondary data published in databases.

Another study reported that health science research has increased in recent years. For instance, Saudi Arabia currently leads the Arab world in medical education and research. According to a bibliometric study conducted in Jordan, an increase in the number of research articles and international collaborations was reported from years 2008 to 2017 [13]. An analysis of the distribution by subject area reveals that the area of medicine has the highest number of publications compared to other areas in the health sciences. This implies that these other areas need more attention from politicians and researchers to achieve a global research approach [13].

This study only analysed the number of manuscripts, quality, and impact of publication citations. Furthermore, it was limited to scientific databases such as Scopus. Future research should compare the productivity of health research with other high-income, scientifically advanced countries [8–12].

For example, in Peru, there are a few studies that measure the thesis rate of medical postgraduate students in the Doctorate program for Health Sciences in Lima. A study by Fernandez-Giusti et al. mentioned that the highest thesis defense production took place between 2014 and 2020. Additionally, there was an enhanced relationship between the students' doctoral thesis defense and their career doctorate [14]. Other studies have shown that the academic production of public and private Peruvian colleges has increased notably, especially since 2014. This could be a result of greater investment, further incentives, improvement of laboratories, and hiring of researchers with extensive experience, among other reasons [15].

the vice-chancellors of four universities (14.2%) had published at least one article in Scopus. Therefore, the scientific production of the research vice-rectors of the universities that were evaluated was deficient. This can be identified using bibliometrics [16]. Therefore, it is also important to mention that the licensing process that the human medicine schools in Peru have to follow drives the current and constant increase in scientific publications. Similarly, interinstitutional collaboration with other national and foreign universities produces quality information [17].

The main limitation to the present study was that it was only possible to show the scholarly output of the Peruvian medical schools in the Scopus database, although future studies could be carried out to evaluate other databases such as Web of Science, Medline, and Embase. Another limitation was that only a specific period (2015–2020) was evaluated, although it is predicted that the highest production peak is expected in the years following 2020.

This research will help determine the scientometric profile of the academic production of all Peruvian medical schools in Scopus. It will also help in the choice of journals, research fields, and topics. Because the main strength of a scientometric study is to provide a statistical analysis of the scientific publications of the Peruvian medical schools.

## 5. Conclusions

There has been a notable and constant increase in the scholarly output of the Peruvian medical schools from 2015 to 2020. Additionally, it was found that medical publications contribute the most to the total scientific production of Peru. This coincides with the enactment of a new university law that, among other aspects, encourages research in Peru.

## Figures and Tables

**Table 1:** All faculties of health of sciences or medicine in Peru.

*N* ^*°*^	Scopus ID	University	*N* ^*°*^	Scopus ID	University
1	60071237	Universidad Peruana Cayetano Heredia	22	60105266	Universidad Nacional San Luis Gonzaga de Ica
2	60071231	Universidad Nacional Mayor de San Marcos	23	60113141	Universidad Privada San Juan Bautista
3	60103706	Universidad Peruana de Ciencias Aplicadas	24	60109730	Universidad Católica Los Ángeles de Chimbote
4	60089535	Universidad Nacional San Agustín de Arequipa	25	60105265	Universidad Nacional Toribio Rodríguez de Mendoza de Amazonas
5	60078122	Universidad Científica del Sur	26	60105267	Universidad Nacional Micaela Bastidas de Apurímac
6	60104616	Universidad de San Martín de Porres	27	60105305	Universidad Peruana Unión
7	60104719	Universidad San Ignacio de Loyola	28	60105284	Universidad Nacional Hermilio Valdizán
8	60071256	Universidad Nacional San Antonio Abad del Cusco	29	60112059	Universidad Norbert Wiener
9	60108878	Universidad Continental	30	60105109	Universidad Nacional San Cristóbal de Huamanga
10	60071251	Universidad Nacional de Trujillo	31	60110655	Universidad Nacional de Tumbes
11	60078117	Universidad Privada del Norte	32	60071259	Universidad Nacional de Cajamarca
12	60105261	Universidad Ricardo Palma	33	60109322	Universidad Católica Santo Toribio de Mogrovejo
13	60071261	Universidad Nacional Federico Villarreal	34	60105303	Universidad Nacional Jorge Basadre Grohmann
14	60105306	Universidad César Vallejo	35	60089536	Universidad Nacional Santiago Antúnez de Mayolo
15	60105309	Universidad Católica de Santa María	36	60105304	Universidad Peruana los Andes
16	60105301	Universidad Privada Antenor Orrego	37	60112678	Universidad Privada de Tacna
17	60071253	Universidad Nacional de la Amazonía Peruana	38	60105286	Universidad Nacional Amazónica de Madre de Dios
18	60071260	Universidad Nacional del Altiplano de Puno	39	60105328	Universidad Nacional José Faustino Sánchez Carrión
19	60105110	Universidad Nacional del Centro del Perú	40	60105262	Universidad Nacional del Santa
20	60105282	Universidad Nacional Pedro Ruiz Gallo	41	60105285	Universidad Nacional Daniel Alcides Carrión
21	60104034	Universidad Nacional de Piura	42	60120619	Universidad San Pedro

**Table 2:** Scientific production of the Peruvian Medicine Faculties by sub-subject area.

Subcategory	Scholarly output	%	Citations	Authors	Citations per publication	Field-weighted citation impact
Faculties of Medicine in Peru	5406	100	43249	5983	8	1.1
Clinical medicine	3018	56	23332	3388	7.7	1.0
Basic medicine	1927	36	22103	2682	11.5	1.4
Health sciences	1861	34	8506	2709	4.6	0.8
Other medical science	802	15	13315	1311	16.6	2.2
Medical biotechnology	61	1	131	147	2.1	0.3

**Table 3:** Scientific production of all medical faculties in Peru.

Peruvian medical schools	2015	2016	2017	2018	2019	2020	Overall
Universidad Peruana Cayetano Heredia	304	343	349	338	373	465	2172
Universidad Nacional Mayor de San Marcos	103	110	149	163	165	285	975
Universidad Peruana de Ciencias Aplicadas	114	131	99	126	136	99	705
Universidad de San Martín de Porres	40	83	76	92	92	124	507
Universidad Científica del Sur	35	46	24	52	97	227	481
Universidad San Ignacio de Loyola	1	4	17	34	90	148	294
Universidad Continental	3	11	24	37	54	77	206
Universidad Ricardo Palma	6	28	13	25	31	44	147
Universidad Nacional de Trujillo	7	22	19	19	33	42	142
Universidad Privada Antenor Orrego	10	27	35	20	18	29	139
Universidad Nacional San Agustín de Arequipa	5	8	16	21	35	41	126
Universidad Nacional Federico Villarreal	6	8	13	16	20	50	113
Universidad Privada del Norte	0	6	25	25	17	34	107
Universidad Nacional San Antonio Abad del Cusco	5	16	13	20	21	22	97
Universidad Nacional “San Luis Gonzaga” de Ica	4	21	21	12	17	13	88
Universidad Católica Los Ángeles de Chimbote	0	12	13	6	16	33	80
Universidad Privada San Juan Bautista	0	5	7	8	20	33	73
Universidad Católica De Santa María	9	8	8	8	12	24	69
Universidad César Vallejo	3	5	7	7	12	30	64
Universidad Nacional de la Amazonía Peruana	8	10	4	13	10	17	62
Universidad Nacional de Piura	7	9	11	7	7	10	51
Universidad Nacional Pedro Ruiz Gallo	6	9	6	7	10	13	51
Universidad Norbert Wiener-UNW	1	2	2	3	11	31	50
Universidad Nacional del Altiplano de Puno	3	4	9	6	11	13	46
Universidad Católica Santo Toribio de Mogrovejo	2	8	9	6	5	14	44
Universidad Nacional Hermilio Valdizán	1	2	1	4	6	29	43
Universidad Peruana Unión	1	1	0	2	10	25	39
Universidad Nacional del Centro del Perú	0	1	3	5	11	17	37
Universidad Peruana los Andes	0	6	1	2	6	19	34
Universidad Nacional de San Cristóbal de Huamanga	2	1	3	3	13	11	33
Universidad Nacional de Cajamarca	2	5	2	2	7	3	21
Universidad Privada de Tacna	0	1	2	6	5	5	19
Universidad Nacional Toribio Rodríguez de Mendoza de Amazonas	0	0	0	0	4	12	16
Universidad Nacional de Tumbes	1	0	2	1	8	3	15
Universidad Nacional Santiago Antúnez de Mayolo	0	0	3	3	3	3	12
Universidad Nacional José Faustino Sánchez Carrión	0	1	2	1	3	2	9
Universidad Nacional Jorge Basadre Grohmann	0	1	0	1	3	2	7
Universidad Nacional Micaela Bastidas de Apurímac	1	0	3	2	0	0	6
Universidad Nacional Amazónica de Madre de Dios	0	1	0	1	0	2	4
Universidad Nacional Daniel Alcides Carrión	0	2	0	0	1	1	4
Universidad Nacional del Santa	0	0	0	1	1	2	4
Universidad San Pedro	0	1	0	1	0	0	2
Total	944	1085	1184	1267	1431	2039	7950

**Table 4:** Top 10 scientific journals of the scientific production of the medical schools of Peru.

Scopus source	Publications	Citations	Authors	Citations per publication	CiteScore 2019	SCImago Journal Rank (SJR)
*Revista Peruana de Medicina Experimental y Salud Publica*	500	1017	997	2.0	1.1	0.2
American Journal of Tropical Medicine and Hygiene	130	931	305	7.2	4.0	1.1
*Revista de Gastroenterología del Perú*	115	83	278	0.7	0.4	0.1
PLoS Neglected Tropical Diseases	82	1063	188	13.0	7.6	2.1
Medwave	75	81	197	1.1	0.6	0.1
Journal of Oral Research	63	31	101	0.5	0.5	0.1
*Revista Medica de Chile*	41	37	67	0.9	0.9	0.2
*Salud Publica de Mexico*	39	46	73	1.2	2.3	0.6
*Revista Habanera de Ciencias Medicas*	39	15	105	0.4	0.3	0.1
*Revista Chilena de Infectología*	34	44	80	1.3	0.7	0.2

**Table 5:** Top 10 most productive authors in Peruvian medicine.

Name	Affiliation	Scholarly output	Citations	Citations per publication	Field-weighted citation impact	*h*-index
Miranda, Juan Jaime	Universidad Peruana Cayetano Heredia	213	9363	44.0	5.5	41
Bernabe-Ortiz, A.	Universidad Peruana Cayetano Heredia	126	7159	56.8	7.1	27
Mejia, Christian R.	Universidad Continental	110	283	2.6	0.4	12
Gilman, Robert Hugh	Johns Hopkins University	94	985	10.5	1.0	80
García, Héctor Hugo	Georgetown University	93	1066	11.5	1.0	49
Hernandez, Adrian V.	Duke University	86	2632	30.6	2.8	48
Dominguez-Lara, Sergio Alexis	Universidad San Martin de Porres	81	203	2.5	1.8	9
Taype-Rondán, Álvaro	Universidad Peruana Cayetano Heredia	79	221	2.8	0.7	9
Gotuzzo, Eduardo H.	Universidad Peruana Cayetano Heredia	71	774	10.9	0.9	55
Carrillo-Larco, Rodrigo M.	Universidad Peruana Cayetano Heredia	63	1279	20.3	2.9	15

## Data Availability

The data used in this bibliometric study will be available upon authorization of the corresponding author.
